# TUT7-Mediated Uridine Degradation of MCPIP1 in the Pterygium to Regulate TRAF6-Mediated Autophagy

**DOI:** 10.1167/iovs.66.4.41

**Published:** 2025-04-16

**Authors:** Juanjuan Li, Hao Ji, Yanze Xu, Weijia Zhang, Yuru Yin, Yubing Zhao, Yan Du, Anni He, Dandan Zhao

**Affiliations:** 1 Department of Ophthalmology, Yan'An Hospital of Kunming City, Kunming, Yunnan, China; 2 Department of Information, The First People's Hospital of Yunnan Province, The Affiliated Hospital of Kunming University of Science and Technology, Kunming, Yunnan, China

**Keywords:** pterygium, MCPIP1, TRAF6-BECN1, autophagy, TUT7

## Abstract

**Purpose:**

Pterygium is a prevalent ocular disorder characterized by the proliferation of fibrovascular tissue beneath the conjunctiva. The precise role of monocyte chemotactic protein-induced protein 1 (MCPIP1) in the pterygium remains elusive.

**Methods:**

Immunohistochemistry, Western blot, and quantitative RT-PCR were used to analyze the expression of MCPIP1 and other regulators. The role of MCPIP1 in pterygium fibrosis was assessed both in vitro and in vivo. Further, Co-immunoprecipitation and ubiquitination assays were performed to investigate the impact of MCPIP1 on the TRAF6-BECN1 signaling pathway. The role of MCPIP1 in autophagy regulation was studied through immunofluorescence experiments, while transwell migration and wound-healing assays were employed to assess the migratory and proliferative capabilities of human pterygium fibroblast (HPF) cells. Additionally, in vitro transcription and uridylylation experiments provided mechanistic insights into the regulatory role of terminal uridyltransferase 7 (TUT7) on MCPIP1 mRNA.

**Results:**

The results showed that MCPIP1 negatively regulates the fibrosis and autophagy of HPF cells, thereby inhibiting the development of pterygium. In terms of its mechanism, MCPIP1 facilitated the assembly of the TRAF6-BECN1 complex, augmented BECN1 ubiquitination, induced autophagy, and attenuated cell migration and proliferation abilities while suppressing HPFs’ cell fibrosis. The function of MCPIP1 was weakened by TUT7, which reduced the stability of MCPIP1 mRNA and thus alleviated the negative regulatory effect of MCPIP1 on pterygium.

**Conclusions:**

In summary, the current study revealed that MCPIP1 promotes autophagy by positively regulating the TRAF6-BECN1 signaling pathway, thereby suppressing pterygium development. Conversely, TUT7 uridylylation modulated MCPIP1’s regulation of pterygium.

Pterygium is a condition characterized by the growth of fibrous vascular tissue from the conjunctiva into a wedge-shaped extension that firmly adheres to the cornea within the interpalpebral fissure.^1^ This condition is notably prevalent among outdoor workers and elderly individuals in China, affecting approximately 13.4% of people over 40 years old (equivalent to nearly 109 million individuals).^2^ Pterygium management varies based on its activity level; quiescent cases require regular observation, whereas rapidly progressing instances necessitate surgical intervention despite an inherent risk for recurrence due to its characteristic nature.^2^ A comprehensive understanding of its pathogenesis is essential for advancing treatment strategies aimed at reducing recurrence rates. While the precise pathogenesis of pterygium remains unclear, current research indicates that fibrosis plays a pivotal role. The abnormal proliferation and migration of fibroblasts highly contribute to pterygium progression. Studies have demonstrated that conjunctival injury resulting from trabeculectomy stimulates fibroblast proliferation, leading to the formation of fibrotic scars and subsequent development of pterygium.^3^ Additionally, pterygium tissue exhibits elevated α–smooth muscle actin (SMA) and extracellular matrix deposition, both hallmark features of fibrosis.^4^ Therefore, targeting fibrosis in pterygium progression represents a promising approach to developing novel therapeutic strategies.

Autophagy, a crucial cellular degradation pathway, is essential in maintaining intracellular homeostasis by facilitating the breakdown of damaged organelles and misfolded proteins through the lysosomal system.^5^ Autophagy serves various physiological functions, including the clearance of intracellular components, regulation of cell death, developmental processes, and antiaging mechanisms. Moreover, it extensively participates in diverse physiological responses such as cancer progression, metabolism modulation, and cardiovascular diseases.^6^^,^^7^ Autophagosome formation involves the encapsulation of cytoplasmic proteins and dysfunctional organelles, followed by fusion with the lysosomal membrane to release autophagic bodies into the lysosomal lumen, where these macromolecular components are subsequently degraded or processed.^8^ The TNF receptor associated factor 6 (TARF6) functions as an E3 ubiquitin ligase that facilitates the ubiquitination of beclin 1 (BECN1), a critical regulator for autophagy initiation and progression.^9^ The BECN1 protein, in conjunction with PIK3C3 and PIK3R4, forms the class III PtdIns3K complex that specifically regulates cellular autophagy.^8^

Recent studies have highlighted the involvement of autophagy in the regulation of diverse fibrotic disorders, including renal fibrosis,^10^^,^^11^ liver fibrosis,^12^ and myocardial fibrosis.^13^ Both the upregulation and downregulation of autophagy have been implicated in the development of fibrotic lesions, suggesting a complex relation between autophagy and fibrosis. Downregulating autophagy has been shown to promote fibrosis,^14^^–^^16^ and experimental evidence indicates that the augmented autophagy contributes to the progression of renal interstitial fibrosis, reinforcing the notion that the upregulation of autophagy also promotes fibrotic processes.^11^ Therefore, autophagy may play a crucial role in the pathogenesis of pterygium fibrosis. The current research suggests that autophagic dysfunction promotes fibrosis and pterygium occurrence by activating the SQSTM1–PKCι–NF-κB signaling pathway.^4^ However, the precise role of autophagy in the pterygium pathogenesis remains elusive.

The ZC3H12A gene encodes monocyte chemotactic protein-induced protein 1 (MCPIP1), which was initially identified in human peripheral blood mononuclear cells induced by MCP-1.^17^ MCPIP1 is involved in regulating inflammation, particularly in response to proinflammatory factors such as IL-6 and lipopolysaccharide (LPS).^18^ While the role of MCPIP1 in fibrosis is well documented, there is a lack of definitive research regarding its involvement in pterygium. In this study, we demonstrated that MCPIP1 acts as a crucial link in the TRAF6-BECN1 signaling pathway, inducing autophagy activation and subsequently suppressing the proliferation and fibrosis of pterygium. These findings suggest that targeting MCPIP1 may hold promising therapeutic potential in pterygium treatment and in preventing its recurrence.

## Materials and Methods

### Animals

Six-week-old BALB/c nude mice were purchased from the Hu Nan Animal Centre and housed under specific pathogen-free (SPF) conditions. The environment was maintained at a temperature of 25°C, with 60% humidity and a 12-hour light/dark cycle. The animal study was approved by the Ethics Committee of Yan'An Hospital of Kunming City and was conducted adhering to ethical guidelines (approval number: 2023019, laboratory animal license number: SYXK(Dian)K2020-004).

Following a 1-week acclimation period, the 18 athymic nude mice were randomly allocated into three experimental groups (*n* = 6 per group). In the first group, the right eye of each mouse was injected with 1 × 10^4^ human pterygium fibroblasts, suspended in 5 µL matrix gel through the nasal subconjunctival gap, while the left eye received an equal amount of matrix gel in the corresponding area as a control. In the second group, the right eye of each mouse was injected with fibroblasts stably overexpressing MCPIP1 at a concentration of 1 × 10^4^ cells, suspended in matrix gel, while the left eye was injected with an equal number of fibroblasts transfected with an empty vector. The nasal subconjunctival gap of the right eye of the third group was injected with a mixture containing 1 × 10^4^ stable knockdown MCPIP1 fibroblasts and matrix gel, while the left eye received an equal number of fibroblasts transfected with an empty vector. After injection, eyes were regularly observed for pterygium development, and photographs were captured using a microscope on days 0, 3, 7, and 14. On day 14, mice were euthanized using CO_2_, followed by tissue fixation in a 4% paraformaldehyde solution. The euthanasia of mice was performed in accordance with the American veterinary medical association (AVMA) Guidelines for the Euthanasia of Animals.^19^

An additional set of 24 BALB/c nude mice (6–8 weeks old) was randomly assigned to four groups (*n* = 6 per group). The mice were subcutaneously injected with 1 × 10⁷ human pterygium fibroblast (HPF) cells, either with MCPIP1 overexpression, MCPIP1 knockdown, or their respective controls. Tumor length and width were measured weekly using a Vernier caliper, and the tumor volume was calculated using the following formula: volume = (length × width²)/2, where “length” is the longest diameter of the tumor, and “width” is the longest transverse diameter perpendicular to this longest diameter. After 4 weeks, all mice were euthanized as described above, and the xenograft tumors were excised, weighed, and processed for further analysis.

### Isolation and Cultivation of Primary HPFs, Human Normal Conjunctival Fibroblasts, and the Cultivation of Human Foreskin Fibroblasts and HeLa Cells

All human tissue samples were collected following approval by the Institutional Review Board of Yan’an Hospital Affiliated to Kunming Medical University. Written informed consent was obtained from each participant after a thorough explanation of the study’s purpose and procedures, in accordance with the principles of the Declaration of Helsinki. Pterygium surgeries were conducted between March 28, 2024, and August 8, 2024. Pterygium tissues were harvested from 10 patients diagnosed with primary pterygium during these surgical procedures. Normal conjunctival tissues were obtained from the loose bulbar conjunctiva above or below the eyes of 10 patients who underwent scleral buckling surgery for retinal detachment. The inclusion criteria for participants were (1) no history of ocular diseases or prior ocular surgeries, (2) no history of long-term use of topical ophthalmic medications, and (3) absence of systemic conditions such as infectious diseases, immunodeficiency disorders, or diabetes mellitus. Detailed patient information is provided in Supplementary Table S1.

Following the surgical procedures, the collected pterygium and conjunctival tissue samples were placed in Advanced Dulbecco's Modified Eagle Medium/Ham's F-12 (DMEM/F-12; Gibco Life Technologies, Karlsruhe, Germany), supplemented with 10% fetal bovine serum (FBS; Gibco Life Technologies), 100 U/mL penicillin, and 100 µg/mL streptomycin (Gibco Life Technologies). These samples were transported on ice and processed within 2 hours. In a sterile environment, the tissue samples were washed three times with PBS (Gibco Life Technologies), cut into small fragments (approximately 1 × 2 mm), and digested in 0.25% trypsin-EDTA (Gibco Life Technologies) at 37°C for 15 minutes. The digestion mixture was then diluted with culture medium, passed through 70-µm cell strainers (BD Falcon, Franklin Lakes, NJ, USA), and centrifuged at 200 *g* for 5 minutes. The resulting pellet was resuspended in the culture medium, inoculated into 12-well cell culture plates, and incubated at 37°C in a humidified atmosphere containing 5% CO_2_. The culture medium was replaced every 2 days, and experiments were performed with cells between three and six passages.

Human foreskin fibroblasts (HFFs) obtained from the Stem Cell Bank, Chinese Academy of Sciences (Shanghai, China), were used as positive controls (standard fibroblasts), while HeLa cells served as negative controls (standard epithelial cells). HFFs were cultured in DMEM/F-12 supplemented with 20% FBS, 100 U/mL penicillin, and 100 µg/mL streptomycin. HeLa cells were maintained in high-glucose DMEM (4.5 g/L; Gibco Life Technologies) containing 10% FBS, 100 U/mL penicillin, and 100 µg/mL streptomycin in a humidified atmosphere of 5% CO_2_ at 37°C.^20^

### Histopathologic Analysis

Pterygium and control conjunctival tissues from a mouse model of pterygium were fixed in 4% paraformaldehyde at 4°C overnight. The fixed tissues were then sectioned into 5-µm slices and stained with hematoxylin and eosin (H&E) solution according to the manufacturer's guidelines (Nanjing Jiancheng, Jiangsu, China). Following staining, the sections were mounted using neutral resins, and images were captured using a light microscope (Olympus, Tokyo, Japan). For the immunohistochemical analysis, the tissue sections underwent deparaffinization, rehydration, and a 10-minute incubation with hydrogen peroxide. The sections were then blocked with 5% normal goat serum and incubated overnight at 4°C with primary antibodies: MCPIP1 rabbit polyclonal antibody (1:100, rabbit pAb, SAB2701079; Sigma-Aldrich, St. Louis, MO, USA), α-SMA (1:50, rabbit pAb, ab5694; Abcam, Cambridge, MA, USA), fibronectin (FN; 1:50, rabbit pAb, ab2413; Abcam), VEGF (1:50, rabbit pAb, 9003-1-AP; Proteintech, Chicago, IL, USA), and Ki67 (1:500, rabbit pAb, ab15580; Abcam). After incubation, the sections were treated with biotinylated secondary antibody for 2 hours at room temperature. Images were then acquired using an Olympus BX61 microscope. Tissues were classified and scored based on staining intensity as follows: (1) negatively stained, score of 0; (2) mildly stained, score of 1; (3) moderately stained, score of 2; and (4) strongly stained, score of 3. The staining intensity index was calculated by dividing the total score by the number of cells.^4^

### Western Blot Assay

The total protein concentration was determined using a bicinchoninic acid protein assay kit (Pierce, Rockford, IL, USA). Individual protein samples were subjected to SDS-PAGE and then transferred onto polyvinylidene fluoride membranes (Millipore, Billerica, MA, USA). The membranes were blocked for 1 hour at room temperature with 5% skimmed milk prepared in 0.05% Tween-20 in TBST buffer, followed by an overnight incubation at 4°C with the primary antibodies. After washing, the membranes were incubated with secondary antibodies conjugated to horseradish peroxidase at room temperature. Bound antibodies were visualized using an enhanced chemiluminescence Western blot detection reagent (Amersham, Buckinghamshire, UK) and captured on film (Kodak, Tokyo, Japan). All experiments were conducted in triplicate. Densitometry analysis was performed using the ImageJ software (National Institutes of Health, Bethesda, MD, USA). The antibodies used in this study were as follows: MCPIP1 (1:1000, rabbit pAb, ab230502; Abcam), α-SMA (1:50, rabbit mAb, SAB5500002; Sigma-Aldrich), FN (1:3000, rabbit pAb, ab2413; Abcam), vimentin (1:1000, rabbit mAb, ab92547; Abcam), pan-keratin (1:50,000, rabbit pAb, 26411-1-AP; Proteintech), β-actin (1:500, mouse mAb, ab8226; Abcam), LC3B (1:400, rabbit mAb, ZRB100; Sigma-Aldrich), SQSTM1 (1:10,000, rabbit mAb, ab109012; Abcam), TRIM59 (1:500, rabbit mAb, ab69639; Abcam), GAPDH (1:500, rabbit mAb, ab8245; Abcam), TUT7 (1:1000, rabbit mAb, 46970; Cell Signaling Technology), BECN1 (1:500, rabbit mAb, HY-P80568; MedchemExpress, Monmouth Junction, NJ, USA), and PIK3C3 (1:1000, rabbit mAb, ab124905; Abcam).

### Immunofluorescence Microscopy

HPFs and human normal conjunctival fibroblasts (HConFs) were seeded onto precleaned coverslips and cultured for 24 hours. The medium was then replaced with DMEM/F-12 containing 1% FBS, and after 48 hours of treatment, the cells were washed three times with PBS and fixed in 4% formaldehyde for 20 minutes. Following another set of three PBS washes, the cells were permeabilized with 0.5% Triton X-100 (Sigma-Aldrich) for 15 minutes and blocked with 10% normal goat serum for 1 hour at room temperature. The cells were then incubated overnight at 4°C with primary antibodies against pan-keratin (1:50, rabbit pAb, 26411-1-AP; Proteintech), vimentin (1:250, rabbit mAb, ab92547; Abcam), and microtubule-associated protein 1 light chain 3 (LC3) (1:1, rabbit mAb, ab192890; Abcam). After rinsing with PBS, the cells were incubated for 2 hours at room temperature with Alexa Fluor 555 Donkey Anti-Rabbit IgG (1:200, ab150074; Abcam). Nuclei were stained with 4′,6-diamidino-2-phenylindole (DAPI) included in the mounting medium. Finally, the coverslips were mounted onto slides using a mounting medium with DAPI (H-1200; Vector, Burlingame, CA, USA), and fluorescent images were captured using a Leica inverted fluorescence microscope (DMi8/DFC 550; Leica, Wetzlar, Germany).

### Gene Knockdown and Overexpression

Small interfering RNA (siRNA) technology was employed to downregulate the expression of the target gene. MCPIP1 and TRAF6 siRNAs were synthesized by Thermo Fisher Scientific (Waltham, MA, USA). A scrambled siRNA (si-NC) with no known target in HPF cells served as a nonspecific control. To generate the MCPIP1 overexpression plasmid, the coding sequence of MCPIP1 was cloned into the pCMV plasmid as described in Han et al.^21^ An empty vector, lacking the MCPIP1 insert, was used as a control. To evaluate the effect of MCPIP1 knockdown and overexpression on LC3B localization and expression, HPF cells expressing GFP–LC3B were transfected with MCPIP1-specific siRNA and the overexpression plasmid.

### Cellular Proliferation and Viability Assessment

Cell proliferation was evaluated using an EDU assay kit (C0071S; Beyotime, Shanghai, China). Briefly, HPFs were exposed to a 10-µM EDU working buffer for 24 hours. After fixation with 4% paraformaldehyde for 15 minutes and permeabilization using PBS containing 0.3% Triton X-100 for 10 minutes, the cells were incubated with 200 µL of click reaction solution for 30 minutes. Images were captured and analyzed using a Leica DMi8 fluorescence microscope and ImageJ software.^22^ To evaluate cell viability, a CCK-8 assay was performed. HPFs were seeded into 96-well cell culture plates at a density of 2 × 10³ cells per well and allowed to adhere for 18 hours before treatment. Subsequently, 10 µL CCK8 solution (CK04; Dojindo, Kumamoto, Japa) was added to each well and incubated at 37°C for 2 hours. Absorbance was measured at 450 nm using a Model 680 Microplate Reader (Bio-Rad, Hercules, CA, USA). All experiments were performed in triplicate and repeated at least three times.

### Transwell Migration and Wound-Healing Assays

For the transwell migration assay, 30,000 cells were resuspended in a serum-free medium and seeded into the upper chamber of a 24-well transwell plate (Corning, Falcon, NY, USA). The lower chamber was filled with a medium containing 10% FBS as a chemoattractant. After migration, the cells were fixed, stained, and counted using a light microscope. For the wound-healing assay, cells were seeded in 6-well plates, and a scratch was introduced using a pipette tip. The wound closure was monitored and photographed at specific time points during incubation in the serum-free medium under different treatment conditions.

### Protein Interaction Prediction

Potential interaction partners of MCPIP1 were predicted using the BioGRID 4.4 database (https://thebiogrid.org/). The identified proteins were then visualized through network analysis using specialized software.^23^

### RNA Purification and Quantitative RT-PCR Analysis

Total RNA was extracted using TRIzol reagent (Invitrogen, Carlsbad, CA, USA; 15596-026). Complementary DNA (cDNA) synthesis was carried out with the PrimeScript RT reagent kit (Takara, Shiga, Japan; RR047A). Quantitative RT-PCR (qRT-PCR) was performed using the SYBR Green Premix Ex Taq II kit (Takara, RR820A) on an ABI ViiATM 7 Real-Time PCR System (Applied Biosystems, Waltham, MA, USA). The relative mRNA expression levels of the target genes were calculated using the comparative Ct method, with GAPDH as an internal control. Primer sequences used for qRT-PCR are listed in Supplementary Table S2.

### mRNA Stability Assay

HPFs were treated with 100 ng/mL LPS for 4 hours, followed by incubation with actinomycin D (5 µg/mL) for the specified time points. Cells were then harvested, and total cellular RNA was extracted for mRNA quantification using qRT-PCR, as previously outlined.^24^ Actinomycin D is rapidly absorbed by cells and preferentially intercalates into GC-rich DNA sequences, forming stable complexes that inhibit transcription by all eukaryotic RNA polymerases.

### Dual-Luciferase Reporter

Cells were cotransfected with the firefly luciferase reporter plasmid, the pRL-TK-*Renilla* luciferase plasmid, and either the TUT7 plasmid or an empty control plasmid. After culturing for 48 hours, cells were collected and lysed. Firefly and *Renilla* luciferase activities were measured using the Dual-Luciferase Reporter Assay System (Promega, Madison, WI, USA; E1910), following the manufacturer's guidelines.

### RNA-Immunoprecipitation

Cells were harvested and lysed using APB lysis buffer supplemented with protease inhibitors and RNase inhibitors. Five percent of the cell lysates were retained as input samples, while the remaining lysates were incubated with the specified antibody and Protein A–conjugated agarose beads for 4 hours at 4°C. The beads were then pelleted and washed three times with APB buffer containing the RNase inhibitor. To assess immunoprecipitation efficiency, 5% of the beads were analyzed by SDS-PAGE. The remaining beads, along with the input samples, were resuspended in 200 µL NucleoZol (Takara, Shiga, Japan) for RNA extraction, which was followed by RT-PCR and qRT-PCR, as previously described. The primer sequences used for RT-PCR and qRT-PCR were MCPIP1–untranslated region (UTR) forward (5′-ATCACAGATAGCGGTCCCCA-3′) and MCPIP1-UTR reverse (5′-GGCAATAGCTTTTTTTTTCTTTTAA-3′).

### RNA Electrophoretic Mobility Shift Assay

Cy3-labeled and unlabeled RNAs were procured from Genomics (New Taipei City, Taiwan). In total, 75 fmol of the synthesized RNAs was incubated with immunopurified TUT7 from HEK293T cells at 37°C for 30 minutes. Following incubation, the RNA samples were combined with the RNA loading dye (1 × TBE, 20% glycerol, 0.05% bromophenol blue) and subjected to separation on a 6% native PAGE. Fluorescence signals from the Cy3-fluorophore or SYBR Green II staining were then detected using the iBright FL1000 Imaging System (Invitrogen).

### In Vitro Transcription and In Vitro Uridylylation

In vitro transcription was carried out using T7 RNA polymerase (Promega) following the manufacturer's guidelines. Briefly, 5 µg of the linearized DNA template was combined with 40 units of T7 RNA polymerase in a 100-µL reaction mixture containing 40 mM Tris (pH 7.9), 6 mM MgCl_2_, 2 mM spermidine, 10 mM NaCl, 10 mM Dithiothreitol (DTT), 2.5 mM NTP mix, and 100 units of RNase inhibitor. The reaction was conducted for 2 hours at 37°C. The resulting RNA transcripts were purified using NucleoZol, precipitated with 100% isopropanol (Sigma-Aldrich), and resuspended in 10 µL RNase-free water. For the in vitro uridylylation assay, 1 µg synthesized RNA was incubated with either wild-type or catalytically inactive TUT7, which had been immunoprecipitated from HEK293T cells transfected with TUT7 expression plasmids, along with α-32P-UTP for 1 hour at 37°C. The resulting RNA samples were then resolved by 1% formaldehyde-agarose gel electrophoresis and visualized using SYBR Green II staining and radiography.

### Immunoprecipitation and Ubiquitination Assay

The immunoprecipitation (IP) and ubiquitination assays were conducted following established protocols. Briefly, HPF cells were transfected with a control vector (mock vector), Myc-tagged TRAF6, Flag-tagged TRAF6, Flag-tagged MCPIP1, Myc-tagged BECN1, or Flag-tagged PIK3C3 using Lipofectamine 2000 (Thermo Fisher Scien-tific Waltham, MA, USA; 11668030). Myc and Flag are widely utilized as epitope tags in molecular biology. As fusion expression tags, they usually do not interact with the target protein and generally do not affect the function or properties of the target protein. Additionally, these tags can be specifically recognized by their respective antibodies, which facilitates the detection and purification of the target protein while minimizing interference from nonspecific proteins. After 38 hours of transfection, the cells were collected, and their lysates were subjected to immunoprecipitation using anti-Flag or anti-Myc antibodies. HPF cells were transfected with a mock vector, Myc-tagged MCPIP1 or Myc-tagged TRAF6, Flag-tagged TRAF6 or Flag-tagged MCPIP1 wild type (WT), and Flag-tagged TRAF6 or Flag-tagged MCPIP1 truncated mutants, using Lipofectamine 2000.

Additionally, HPF cells were transfected with a mock vector, Myc-tagged MCPIP1 or Myc-tagged BECN1, Flag-tagged BECN1 or Flag-tagged MCPIP1 WT, and Flag-tagged BECN1 or Flag-tagged MCPIP1 truncated mutants. After 38 hours of transfection, the cells were collected, and the lysates were subjected to immunoprecipitation using an anti-Myc antibody. The resulting IP complexes were then separated by SDS-PAGE (6%–10%) and analyzed using antibodies against Myc or Flag.

For semi-endogenous IP assays, HPF cells were transiently transfected with Flag-MCPIP1 and subsequently treated with or without 15 µg/mL LPS for 3 hours. The IP assay was conducted using anti-IgG or anti-Flag antibodies, followed by immunoblotting with anti-Flag, anti-TRAF6, and anti-BECN1 antibodies.

For the ubiquitination assay, HPF cells were transfected using Lipofectamine 2000 with a combination of vectors: mock vector, Flag-tagged TRAF6, Myc-tagged BECN1, Flag-tagged BECN1, HA-tagged ubiquitin (Ub), and Flag-tagged MCPIP1. Different concentrations of Myc-tagged MCPIP1 or Flag-tagged TRAF6 were also included. Thirty-eight hours after transfection, the cells were harvested, and the lysates were subjected to immunoprecipitation using either anti-Myc or anti-Flag antibodies. The immunoprecipitated complexes were separated by 6% to 10% SDS-PAGE and then analyzed by immunoblotting with anti-Myc, anti-Flag, and anti-HA antibodies. For the endogenous ubiquitination assay, HPF cells were transiently transfected with a mock vector and an MCPIP overexpression vector, followed by treatment with or without LPS (15 µg/mL) for 3 hours. The IP assay was performed using an anti-TRAF6 or anti-BECN1 antibody, and the resulting complexes were probed with antibodies against TRAF6, BECN1, or ubiquitin.

For the competitive inhibition assays, HPF cells were transfected with either a mock vector or an MCPIP1 overexpression vector and then treated for 6 hours with vehicle (0.1% v/v DMSO), Poly I:C (25 µg/mL), or LPS (15 µg/mL). The resulting cell lysates were subjected to immunoblotting using an anti-LC3A/B antibody, with anti-GAPDH serving as the loading control.

### Statistical Analysis

The data are presented as the mean ± SD, derived from either triplicate samples or measurements from 10 distinct cells. Statistical significance was assessed using either analysis of variance or Student's *t*-test, with all analyses performed using GraphPad Prism 9.0 software (GraphPad Software, San Diego, CA, USA). Results are expressed as mean ± SD from three independent experiments. Statistical significance is indicated in the figures with *P < 0.05, **P < 0.01, ***P < 0.001, and ****P < 0.0001.

## Results

### MCPIP1 Was Expressed at a Low Level in the Fibroblasts of the Pterygium

**Figure 1.**
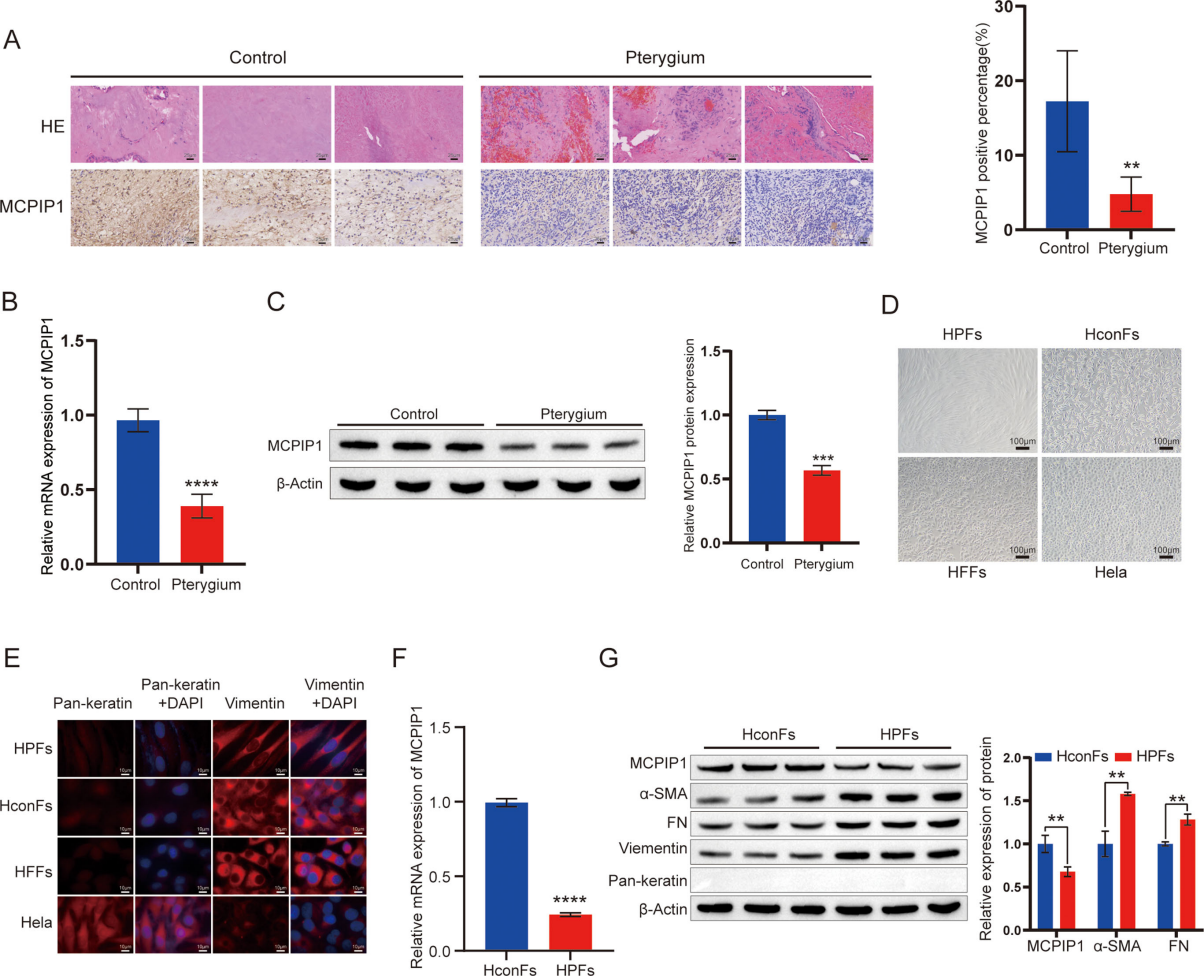
MCPIP1 is expressed at a low level in pterygium fibroblasts. (**A**) A total of 1 × 10^4^ HPFs were coinjected with 5 µL matrix gel into the nasal subconjunctival space in athymic nude mice, while the corresponding area of the left eye received a control injection of 5 µL matrix gel. H&E and IHC staining images of the pterygium mouse model are presented. The quantification results of MCPIP1 protein IHC staining are depicted as bar graphs on the *right*. Mean ± SD; *P < 0.05. *Scale bar*: 25 µm. (**B**) Quantitative analysis of MCPIP1 mRNA expression levels in the pterygium mouse model. Mean ± SD; ***P < 0.001. (**C**) Western blot analysis was employed to determine the protein expression levels of MCPIP1 in the pterygium mouse model. The quantified protein levels are presented using a bar graph (on the *right*). Data are presented as mean ± SD; *P < 0.05. The protein levels were normalized to β-actin as an internal reference. (**D**) Morphological observations of HPFs, HConFs, HFFs, and HeLa using a light microscope. *Scale bar*: 100 µm. (**E**) Representative immunofluorescence images depicting the colocalization of pan-keratin, vimentin, and DAPI in HPFs, HConFs, HFFs, and HeLa cells. *Scale bar*: 10 µm. (**F**) qRT-PCR analysis of MCPIP1 mRNA expression in HConFs and HPFs. Data are presented as mean ± SD; ****P < 0.0001. (**G**) Western blot analysis showing MCPIP1, α-SMAMA, FN, vimentin, and pan-keratin protein levels in HConFs and HPFs isolated and cultured from human pterygium and normal conjunctiva. Quantified protein levels are presented as bar graphs on the *right*; mean ± SD; *P < 0.05, **P < 0.01. The protein levels were normalized to β-actin as an internal reference.

To investigate the role of MCPIP1 in pterygium, we analyzed the GEO dataset GSE155776, which revealed a significant downregulation of MCPIP1 in pterygium tissue (Supplementary Fig. S1A). To further validate, we isolated and cultured HPFs from human pterygium tissue, established a mouse model of pterygium through nasolacrimal injection, and assessed the expression levels of MCPIP1 in the pterygium tissue. The histologic examination using H&E staining showed increased fibroblast count, vascular dilation, and infiltration of immune cells in pterygium tissue compared to controls (Fig. 1A). The immunohistochemical analysis confirmed significantly reduced MCPIP1 expression in pterygium tissue compared to the control (Fig. 1A). Moreover, both the transcriptional and protein levels of MCPIP1 were markedly lower in pterygium than in the control cells (Figs. 1B, 1C).

Fibrosis is commonly employed as one of the parameters for assessing pterygium.^1^ To assess fibrosis, HPFs and HConFs were isolated and cultured from human pterygium tissue and normal conjunctiva, respectively. HFFs and HeLa cells (standard epithelial cells) were employed as positive and negative controls, respectively. Under light microscopy, HPFs, HConFs, and HFFs displayed an elongated spindle-shaped morphology with high cellular transparency, whereas HeLa cells exhibited a characteristic polygonal flattened epithelial cell shape (Fig. 1D). The expression levels of vimentin proteins, a fibroblast marker, were significantly higher in HPFs, HConFs, and HFFs compared to HeLa cells. Consistently, pan-keratin staining was positive in HeLa cells but absent in HPFs, HConFs, and HFFs, confirming the successful isolation of human pterygium and normal conjunctival fibroblasts (Fig. 1E). Subsequent qRT-PCR and Western blot analyses showed a significantly lower expression of MCPIP1 in the HPFs compared with the HConFs (Figs. 1F, 1G). In addition, we assessed extracellular matrix (ECM) remodeling and the accumulation of myofibroblasts, the primary characteristics of pterygium.^25^^–^^27^ Notably, the biomarkers of myofibroblasts (α-SMA) and FN were upregulated in HPFs compared to HConFs (Figs. 1F, 1G). Overall, our findings indicate that MCPIP1 is downregulated in the pterygium tissue, suggesting its potential involvement in the development of fibrosis in pterygium.

### MCPIP1 Impacted Pterygium Fibroblast Fibrosis and Growth

**Figure 2.**
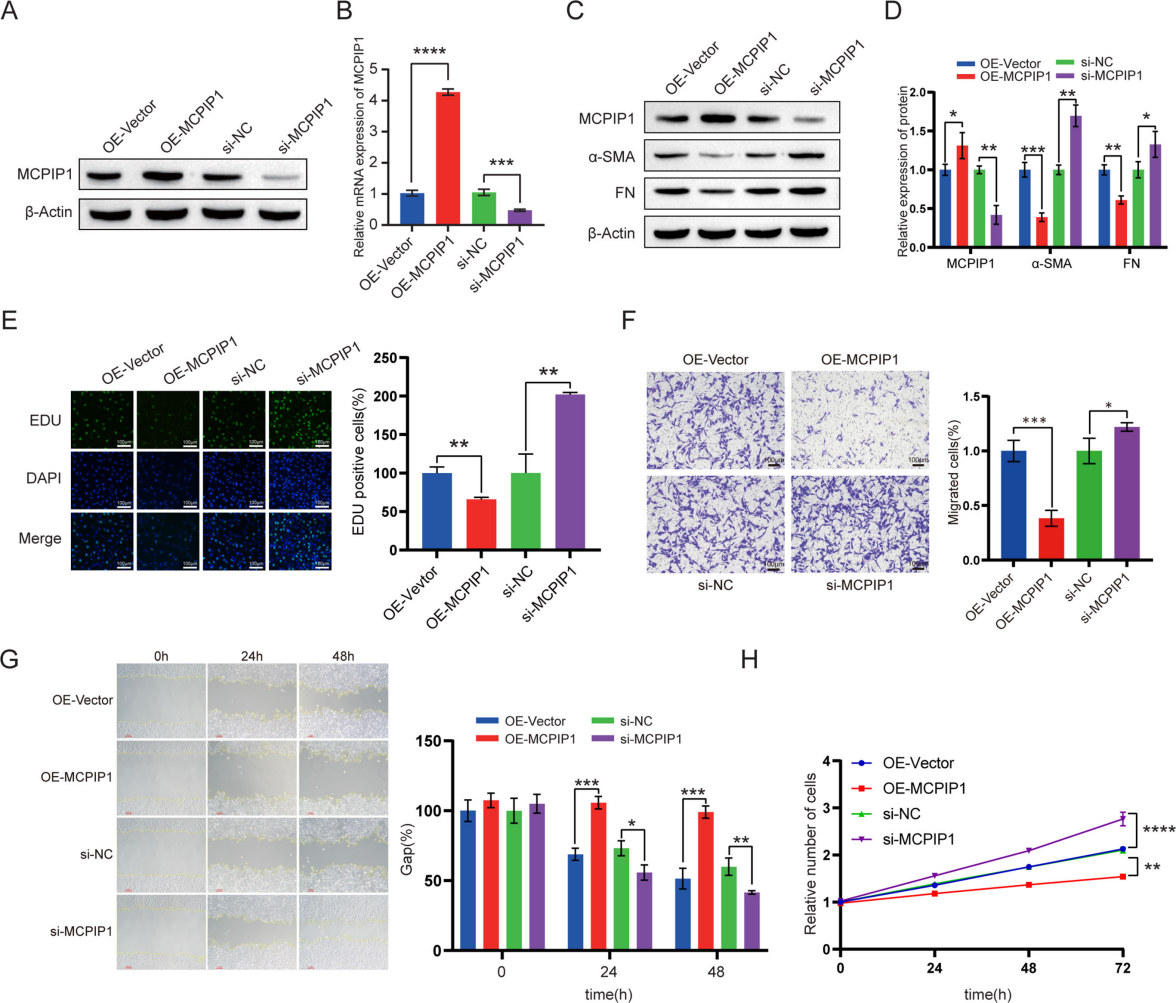
MCPIP1 regulates the fibrosis and growth of pterygium fibroblasts. HPF cells were transfected with OE-Vector, OE-MCPIP1, si-NC, and si-MCPIP1 to establish cell lines with MCPIP1 overexpression and knockdown. Total protein and RNA were subsequently extracted from each cell line. (**A**) Western blot analysis showing MCPIP1 protein levels (anti-MCPIP1). β-Actin was used as an internal reference for normalization. (**B**) MCPIP1 mRNA expression levels were detected by qRT-PCR. Mean ± SD; ***P < 0.001, ****P < 0.0001. (**C**) Representative Western blot images of MCPIP1, α-SMA, and FN protein expression in each cell line, with quantified protein levels displayed as bar graphs. (**D**) Quantification of protein levels from panel **C**. The protein level was normalized to β-actin as an internal reference. Data are presented as mean ± SD; *P < 0.05, **P < 0.01. (**E**) The EDU incorporation assay was used to assess the proliferation of the HPF cell line. DAPI was used for nuclear staining. Data are presented as mean ± SD; ***P < 0.001. (**F**) Transwell migration assay. Cell suspensions were introduced into the upper chamber, and the lower chamber was supplemented with a culture medium. After a 24-hour incubation, cells were fixed and imaged, and the number of migrated cells was quantified (*right*). *Scale ba*r: 100 µm. Data are presented as mean ± SD; *P < 0.05, ***P < 0.001. (**G**) HPF cells were cultivated until confluence, and a scratch was introduced. Representative images of cell migration toward the wound center at specific time points (0, 24, 48 hours) are shown. The wound gap was quantified using ImageJ software and presented as a percentage of the initial gap (*right*). Data are presented as mean ± SD; *P < 0.05, **P < 0.01, ***P < 0.001. (**H**) Cell proliferation was detected using CCK-8 assay at 0, 24, 48, and 72 hours. Data are presented as mean ± SD; **P < 0.01, ****P < 0.0001.

To analyze the role of MCPIP1 on pterygium growth, we constructed overexpressing (OE-MCPIP1) and knockdown (si-MCPIP1) models for MCPIP1 separately (Figs. 2A, 2B). OE-MCPIP1 significantly suppressed the expression of α-SMA and FN proteins, whereas si-MCPIP1 exhibited the opposite effect (Figs. 2C, 2D). In conjunction with the fibrosis marker detection results, these findings substantiate that MCPIP1 negatively regulates fibrosis in the pterygium. Compared with HPFs transfected with an empty vector, overexpression of MCPIP1 significantly suppressed cell proliferation and migration abilities (Figs. 2E, 2F). Similarly, the healing rate of scratch wounds and cell vitality were lower in HPFs transfected with OE-MCPIP1 than those transfected with an empty vector (Figs. 2G, 2H). Conversely, the knockdown of MCPIP1 enhanced the cell migration and proliferation abilities (Figs. 2E, 2H). Overall, MCPIP1 exerted inhibitory effects on the fibrosis and growth processes in HPFs.

### MCPIP1 Inhibited Pterygium Growth In Vivo

**Figure 3.**
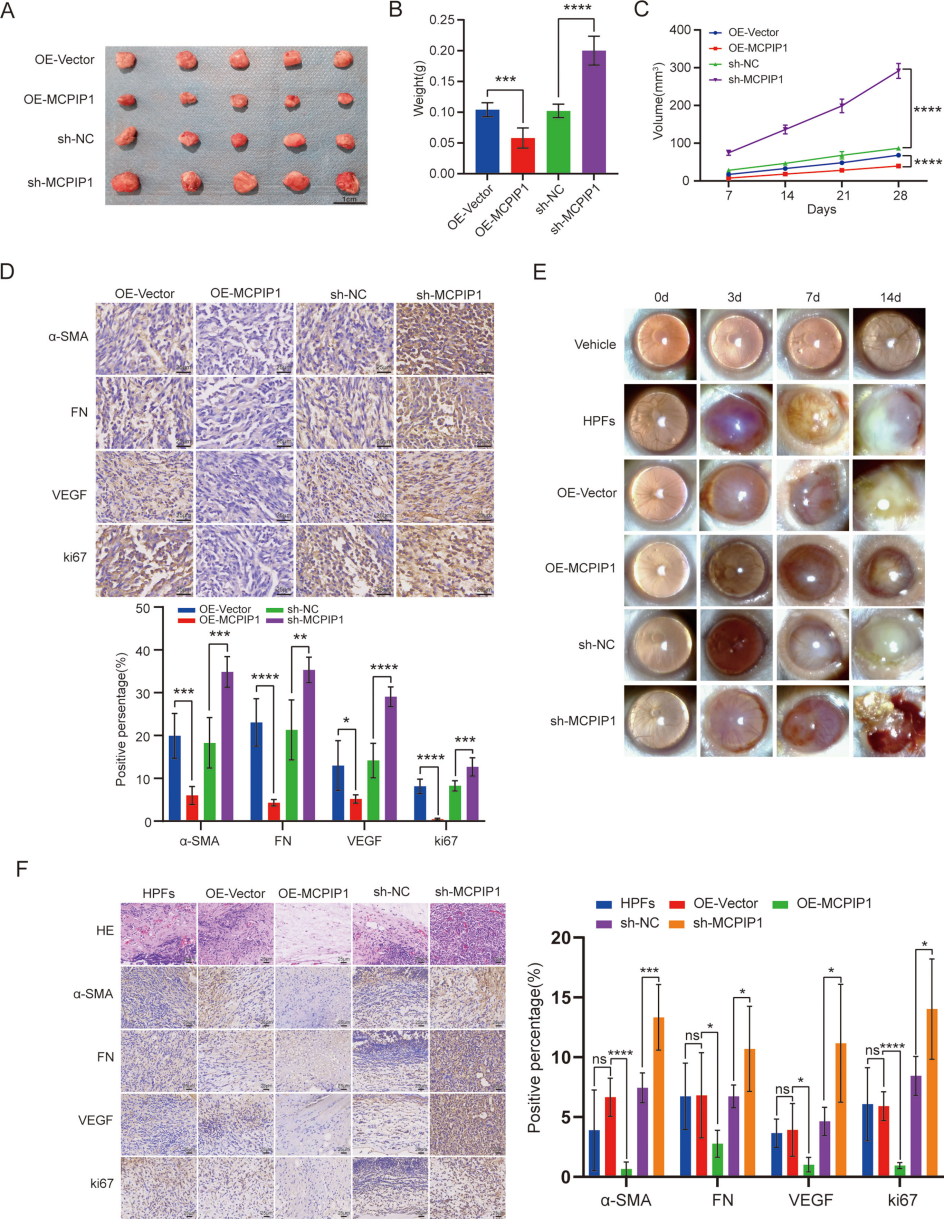
MCPIP1 regulates pterygium growth in vivo. OE-MCPIP1-HPFs, sh-MCPIP1-HPFs, and their respective controls were injected subcutaneously into BALB/c nude mice. On day 28 postinjection, mice were euthanized for tumor morphology assessment. (**A**) Representative images of tumors. (**B**) Data representing tumor weight on day 28. Data are presented as mean ± SD; *P < 0.05, **P < 0.01, ***P < 0.001, ****P < 0.0001. (**C**) Tumor growth curve showing the tumor volume from days 7 to 28, calculated using the formula V = (length × width^2^)/2. Data are presented as mean ± SD; *P < 0.05, **P < 0.01, ***P < 0.001, ****P < 0.0001. (**D**) IHC staining images of α-SMAMA, FN, VEGF, and Ki67 in mouse pterygium tissue, 28 days after subcutaneous injection. Data are presented as mean ± SD; *P < 0.05, **P < 0.01, ***P < 0.001, ****P < 0.0001. *Scale bar*: 25 µm. (**E**) The nasal subconjunctival spaces of athymic nude mice were injected with 1 × 10^4^ HPFs, including OE-MCPIP1, sh-MCPIP1, and their respective control groups. Microscopic images were captured on days 3, 7, and 14. Positive controls consisted of HPFs alone. (**F**) H&E and IHC staining images of α-SMA, FN, VEGF, and Ki67 on day 14 after nasal subconjunctival injection in mice. Data are presented as mean ± SD; ns, not significant; *P < 0.05, **P < 0.01, ***P < 0.001, ****P < 0.0001. *Scale bar*: 25 µm.

The impact of MCPIP1 dysregulation on pterygium growth was further validated through in vivo experiments. OE-MCPIP1-HPFs, sh-MCPIP1-HPFs, and their respective controls were subcutaneously injected into nude mice, and the tumor growth was monitored on day 28 postinjection by measuring its weight and volume. The results showed that OE-MCPIP1 exerted inhibitory effects on tumor growth, whereas sh-MCPIP1 promoted tumor growth in mice (Figs. 3A–C). Pterygium is characterized by proliferation, inflammation, angiogenesis, and ECM deposition.^28^ To investigate the role of MCPIP1 in these processes, immunohistochemistry (IHC) staining was performed on mouse pterygium tissue. The staining showed a significant reduction in the expression of fibrotic factors (α-SMA and FN), VEGF, and cell proliferation marker (Ki67) upon MCPIP1 overexpression. Conversely, silencing of MCPIP1 using sh-MCPIP1 resulted in higher expression of these factors (Fig. 3D), suggesting that MCPIP1 exerts inhibitory effects on the progression of pterygium.

A mouse model of pterygium was successfully established by subconjunctival injection of 1 × 10^4^ HPFs stably expressing OE-MCPIP1/sh-MCPIP1 into nude mice. The success rate of this experiment is 70% to 80%. The criterion for success is that, on day 7 of the experiment, if the pterygium covers 20% to 30% of the mouse corneal area, the model can be deemed successfully established. The lesions were observed and photographed under the microscope on days 0, 3, 7, and 14 postinjection. By day 3, lesion size difference became apparent, with OE-MCPIP1 exhibiting inhibitory effects on pterygium growth and sh-MCPIP1 accelerating its formation (Fig. 3E). Interestingly, a high incidence of ocular hemorrhage was observed in our mouse models (Fig. 3E), which may be attributed to attenuated endothelial cells within the blood vessels, disrupted intercellular connections, basement membrane rupture, and subsequent extravasation of erythrocytes through compromised capillary walls.^29^^,^^30^ Pterygium tissues from each group were collected for H&E and IHC staining to assess fibrosis and growth. The results demonstrated that OE-MCPIP1 suppressed fibrosis, angiogenesis, and cell proliferation in the pterygium tissue, whereas sh-MCPIP1 had a promoting effect (Fig. 3F). Overall, both in vitro and in vivo experiments confirmed that MCPIP1 has an inhibitory role on the fibrosis and growth of pterygium.

### MCPIP1 Interacted With TRAF6 and BECN1

**Figure 4.**
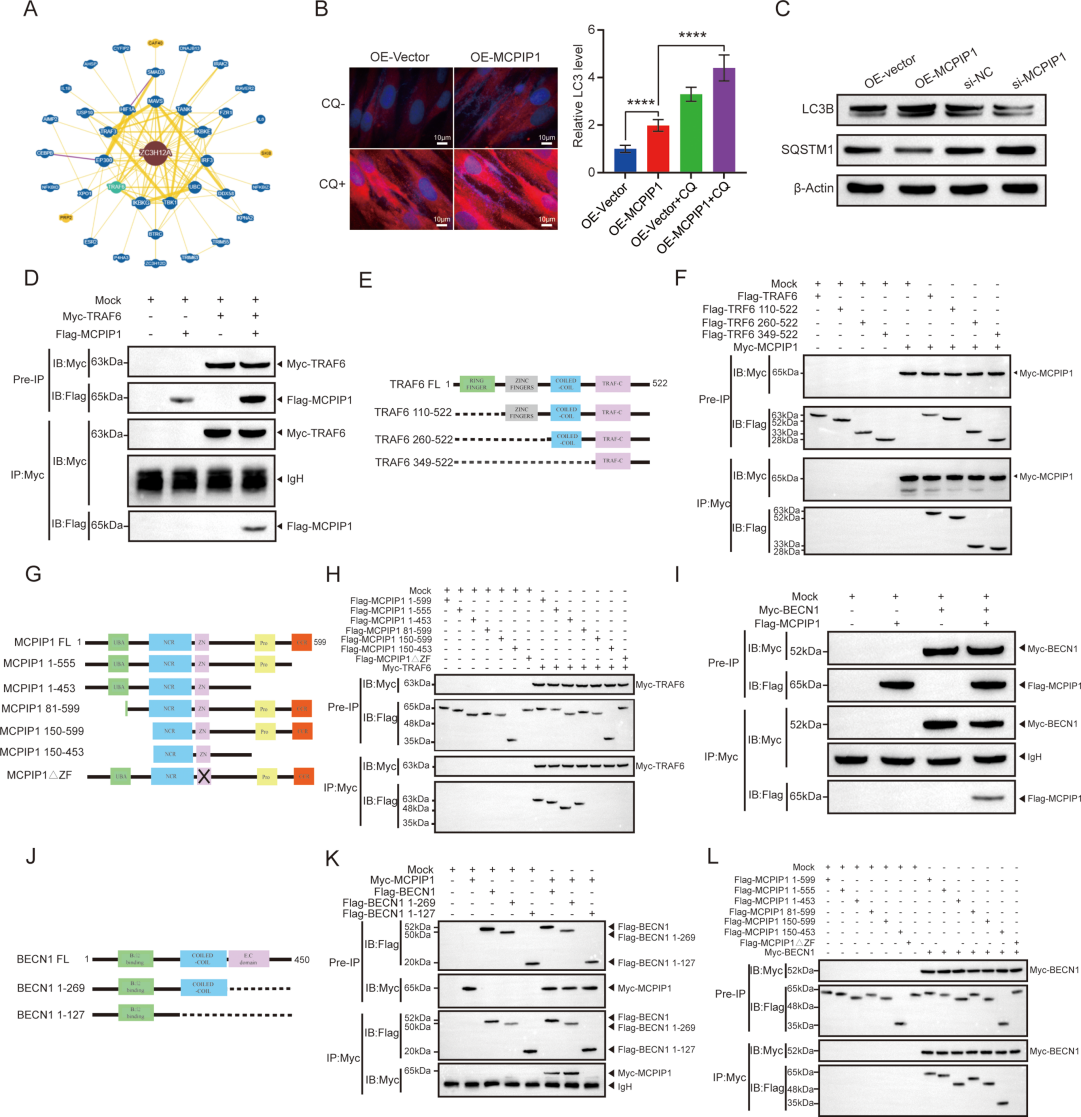
MCPIP1 interacted with TRAF6 and BECN1. (**A**) Schematic representation of the BioGRID website predicting the interaction between MCPIP1 (ZC3H12A) protein. (**B**) HPF cells were transiently transfected with OE-MCPIP1 to establish the stable expression of GFP-LC3. Following a 42-hour incubation, the cells were either treated with 20 µM CQ for 6 hours or left untreated. Fluorescence microscopy was employed for the imaging and subsequent analysis. Cells exhibiting GFP-LC3 puncta were quantified under 200× magnification (*right*). *Scale bar*: 10 µm. Results are presented as mean ± SD; ****P < 0.0001. (**C**) The Western blot data showing the levels of LC3B and SQSTM1 in OE-MCPIP1, siMCPIP1, and corresponding control. The protein levels were normalized to β-actin, used as an internal reference. (**D**) IP experiments were performed using anti-Myc antibody with HPF cells transfected with mock, Myc-TRAF6, and Flag-MCPIP1 constructs. Immunoblotting (IB) analysis was performed using either anti-Myc or anti-Flag antibodies to detect TRAF6 and MCPIP1, respectively. (**E**, **F**) Schematic representation of the full-length TRAF6 and its truncated fragments, highlighting different structural domains (**E**). Full-length TRAF6 or mutant variants containing the specific structural domains were coincubated with Myc-MCPIP1 in HPF cells (**F**). Immunoprecipitation assays were conducted using anti-Myc antibody, followed by immunoblotting with anti-Myc or anti-Flag antibodies. (**G**, **H**) Schematic illustration of the full-length and various truncated fragments of MCPIP1. The UBA domain represents the ubiquitin-associated domain, NCR denotes the N-terminal conserved domain, Pro refers to the proline-rich region, and CCR indicates the C-terminal conserved domain (**G**). Co-IP experiments were carried out by incubating cell lysates containing Flag-MCPIP1 mutants with the Myc-TRAF6 protein (**H**). (**I**) The co-IP assay demonstrating the specific interaction between Myc-BECN1 and Flag-MCPIP1. (**J**, **K**) A schematic diagram illustrating the structure of the full-length BECN1 and its truncated fragments. The full-length or mutant variants were coincubated with Myc-MCPIP1 in HPFs. Immunoprecipitation assays were conducted using an anti-Myc antibody, followed by immunoblotting with anti-Myc or an anti-Flag antibody. (**L**) Co-IP experiments were conducted to assess the interaction between the truncated variants of MCPIP1 and Myc-BECN1.

To elucidate the molecular mechanism underlying MCPIP1’s inhibitory effect of MCPIP1 on pterygium growth, we employed protein interaction prediction tools available on the BioGRID platform and successfully identified TRAF6 as an interacting partner (Fig. 4A). According to reports, MCPIP1 exerts a negative regulatory effect on the inflammatory response by deubiquitinating TRAF6.^31^ In parallel, TRAF6, which functions as an E3 ubiquitin ligase and a scaffold protein, can trigger cellular autophagy via BECN1 ubiquitination.^9^^,^^32^^,^^33^ Consequently, we hypothesized that MCPIP1 potentially modulates the fibrosis process of the pterygium through the autophagy pathway mediated by TRAF6. To investigate this hypothesis, we first assessed the impact of MCPIP1 on cellular autophagy. Notably, the overexpression of MCPIP1 significantly increased the fluorescence intensity of LC3 (Fig. 4B). Additionally, MCPIP1 overexpression resulted in the upregulation of the autophagy marker LC3B and the concurrent downregulation of SQSTM1 expression in HPFs (Fig. 4C). Autophagy is a dynamic process, and the upregulation of LC3 and the presence of autophagosomes may indicate either the initiation or suppression of autophagy.^34^ To further confirm the regulatory role of MCPIP1 in autophagy, cells overexpressing MCPIP1 were treated with the autophagy inhibitor chloroquine (CQ). Compared with untreated OE-MCPIP1 cells, CQ treatment significantly augmented the fluorescence signal of LC3 (Fig. 4B). These findings demonstrate that MCPIP1 promotes autophagy.

The physical interaction between TRAF6 and MCPIP1 was confirmed by co-IP analysis (Fig. 4D). To further identify the specific domains required for this interaction, various TRAF6 truncation mutants were generated, as shown in the figure (Fig. 4E), and subjected to IP with MCPIP1. Results showed that Myc-MCPIP1 interacts with Flag-TRAF6, Flag-TRAF6 110–522, Flag-TRAF6 260–522, and Flag-TRAF6 349–522 (Fig. 4F). Similarly, co-IP using truncated MCPIP1 with TRAF6 revealed that full-length Flag-MCPIP1, Flag-MCPIP1 1–555, Flag-MCPIP1 81–599, and Flag-MCPIP1 1–453 interact with Myc-TRAF6 (Figs. 4G, 4H). This observation suggested that the interaction is mediated by the NCR and ZN domains of MCPIP1 and the TRAF-C domain of TRAF6.

In addition, we observed an interaction between MCPIP1 and BECN1 (Fig. 4I). The co-IP experiments showed that full-length BECN1 and Flag-BECN1 1–269 physically interact with Myc-MCPIP1 (Figs. 4J, JK), while Flag-MCPIP1 1–555, Flag-MCPIP1 81–599, Flag-MCPIP1 1–453, Flag-MCPIP1 150–599, and Flag-MCPIP1 150–453 interact with Myc-BECN1 (Fig. 4L). These results suggest that the coiled-coil domain of BECN1 associates with the Zn domain of MCPIP. In conclusion, MCPIP1 exhibited physical interactions with both TRAF6 and BECN1.

### MCPIP1 Regulated TRAF6 and BECN1 Ubiquitination

**Figure 5.**
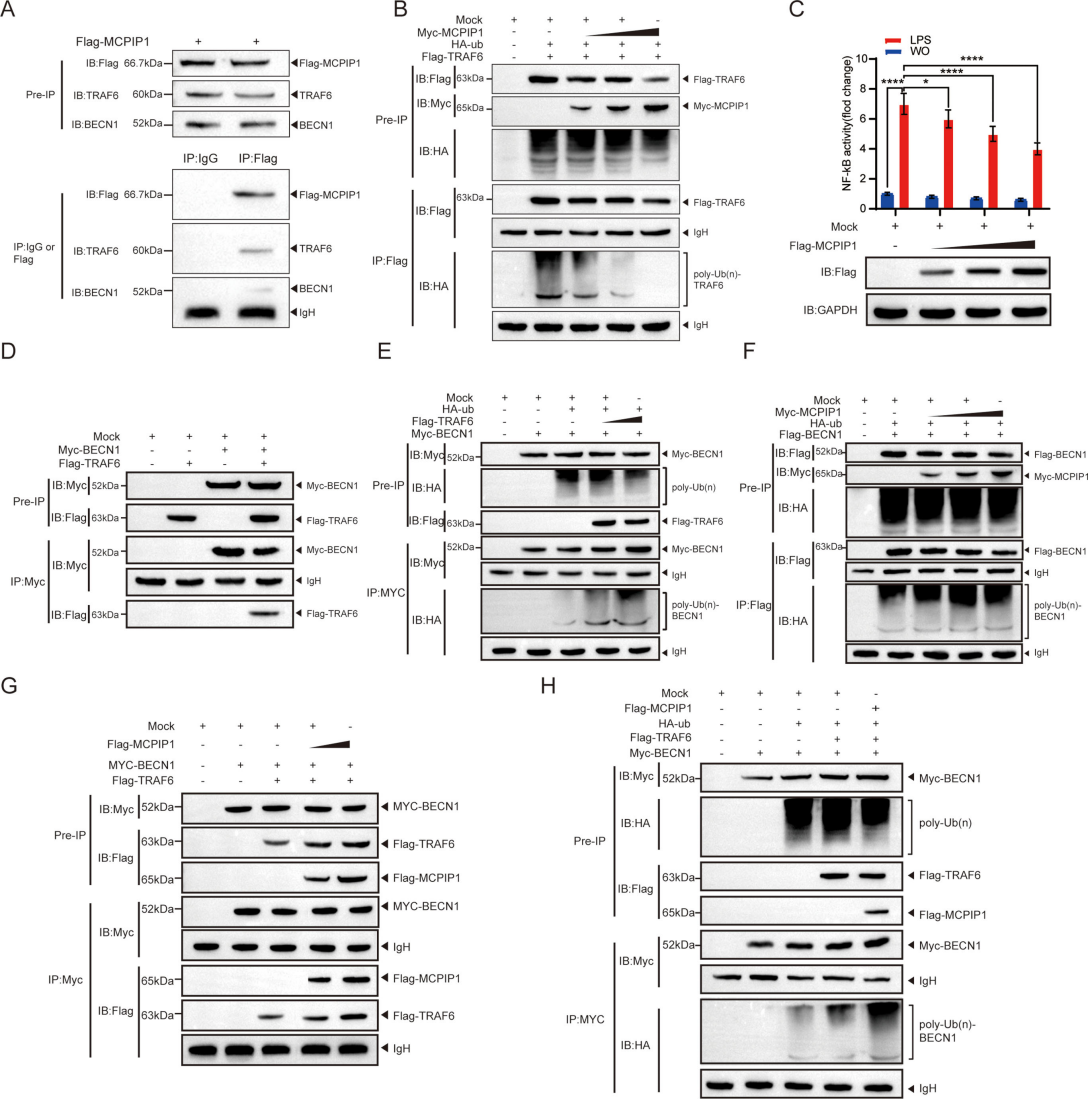
MCPIP1 inhibits TRAF6 ubiquitination and enhances BECN1 ubiquitination. (**A**) HPF cells were transiently transfected with Flag-MCPIP1, followed by semi-endogenous immunoprecipitation using either anti-IgG or anti-Flag antibodies. IB detection was performed using anti-Flag, anti-TRAF6, and anti-BECN1 antibodies. (**B**) HPF cells were transfected with mock, HA-ub, Flag-TRAF6, and varying concentrations of Myc-MCPIP1. The IP experiment was conducted using an anti-Flag antibody, while IB was performed using both anti-Flag and anti-HA antibodies for detection. (**C**) HPF cells were transfected with either mock or varying concentrations of Flag-MCPIP1 and the pBllx-luc NF-κB luciferase vector. The cells were then treated with or without LPS (15 µg/mL) for 6 hours. The expression of Flag-MCPIP1 was detected using anti-Flag antibody, while GAPDH served as a control. Data are represented as mean ± SD; *P < 0.05, ****P < 0.0001. (**D**) Co-IP demonstrating the specific interaction between Myc-BECN1 and Flag-TRAF6. (**E**) The HPF cells were transfected with mock, HA-ub, Myc-BECN1, or varying concentrations of Flag-TRAF6. The IP experiment was conducted using an anti-Myc antibody, and the IP complexes were detected using anti-Myc and anti-HA immunoprobes. (**F**) The HPF cells were transfected with mock, HA-ub, Flag-BECN1, or different concentrations of Myc-MCPIP1. The IP experiment was performed using an anti-Flag antibody, and the complexes were probed with anti-Flag and anti-HA antibodies. (**G**) The HPF cells were transfected with mock, Myc-BECN1, Flag-TRAF6, and different concentrations of Flag-MCPIP1. The IP experiment was performed using an anti-Myc antibody, and the IP complexes were detected using anti-Myc and anti-Flag antibodies. (**H**) The HPF cells were transfected with mock, Flag-MCPIP1, HA-ub, Flag-TRAF6, and Myc-BECN1. The IP experiment was performed using an anti-Myc antibody, and the immunoprecipitated complexes were probed with both anti-Myc and anti-HA antibodies.

To further assess the molecular interaction between MCPIP1 and TRAF6-BECN1, the Flag-MCPIP1 plasmid was transfected into HPF cells, and semi-endogenous immunoprecipitation was performed. The results showed a positive interaction between MCPIP1 and TRAF6-BECN1, with a significant difference when compared to the control IgG (Fig. 5A). TRAF6, through its coiled-coil structure, facilitates self-ubiquitination, activates NF-κB, and ubiquitinates BECN1, thereby inducing autophagy.^9^^,^^32^^,^^35^^,^^36^ Previous studies also indicate that MCPIP1 possesses deubiquitination activity.^31^ Based on this evidence, we examined the impact of MCPIP1 on TRAF6 ubiquitination in pterygium fibroblasts. Our findings demonstrated that MCPIP1 exerts an inhibitory effect on TRAF6 ubiquitination in a concentration-dependent manner (Fig. 5B). In the context of inflammatory response, MCPIP1 deubiquitinates TRAF6 to regulate NF-κB signaling.^31^ Consistently, in HPF cells, with autophagy stimulated by LPS, overexpression of MCPIP1 suppressed the activity of NF-κB, a downstream factor of the Toll-like receptor signaling (Fig. 5C).

We experimentally validated the interaction between TRAF6 and BECN1, along with its capacity to induce the ubiquitination of BECN1 (Figs. 5D, 5E). Given the established interaction between MCPIP1 and BECN1 in previous studies, we conducted further investigations to elucidate the impact of MCPIP1 on BECN1 ubiquitination. Interestingly, MCPIP1 had no impact on the ubiquitination of BECN1, even with increasing concentrations of MCPIP1 (Fig. 5F). Based on the results, we hypothesized that MCPIP1 modulates the ubiquitination of BECN1 by influencing its interaction with TRAF6. Although TRAF6 and BECN1 physically interact in the absence of MCPIP1, the presence of MCPIP1 significantly enhances the interaction in a dose-dependent manner (Fig. 5G). Further, the ubiquitination of BECN1 by TRAF6 was also markedly potentiated in the presence of MCPIP1 (Fig. 5H). These findings suggested that MCPIP1 facilitates the interaction between TRAF6 and BECN1, thereby promoting the ubiquitination of BECN1.

### MCPIP1 Enhanced Autophagy in Pterygium Fibroblasts Via TRAF6

**Figure 6.**
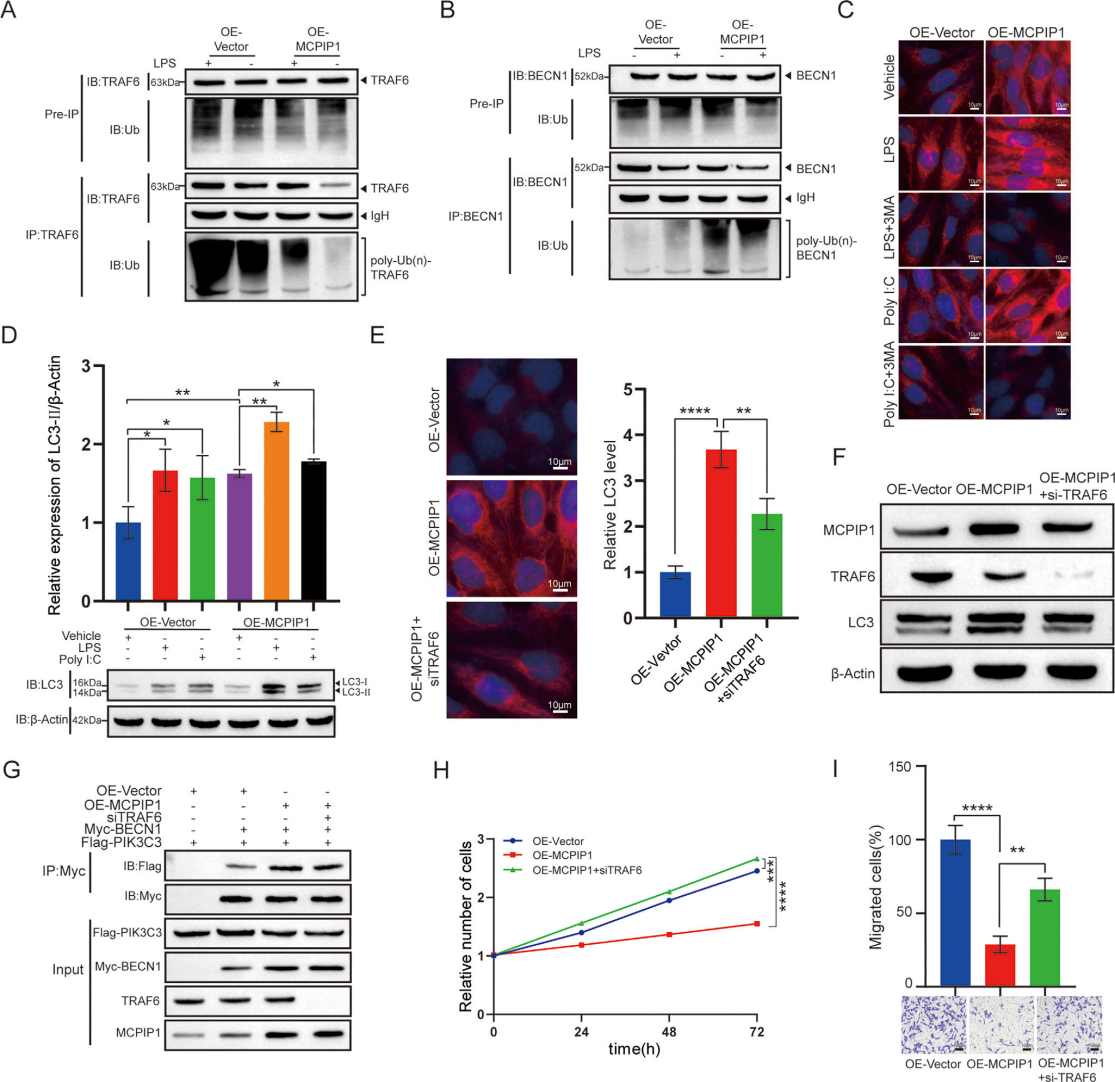
MCPIP1 enhances autophagy in pterygium fibroblasts via TRAF6. (**A**, **B**) The HPF cells transfected with OE-Vector and OE-MCPIP1 were separately treated with or without LPS (15 µg/mL) for 3 hours. Endogenous IP experiments were performed using anti-TRAF6 (**A**) and anti-BECN1 (**B**). The IP complexes were detected using antibodies against TRAF6, BECN1, and Ub. (**C**) HPF cells transfected with OE-Vector and OE-MCPIP1 were treated with DMSO (0.1% v/v concentration), poly I:C (5 µg/mL), 3-MA (5 mM), or LPS (15 µg/mL) for 6 hours. Immunofluorescence staining was performed using anti-LC3, followed by fluorescence confocal microscope imaging. *Scale bar*: 10 µm. (**D**) HPF cells transfected with OE-Vector and OE-MCPIP1 were treated with DMSO, poly I:C, and LPS for 6 hours. Immunoblotting was performed using anti-LC3 and anti–β-actin. Data are presented as mean ± SD; ns, not significant; *P < 0.05, **P < 0.01. (**E**) HPF cells stably expressing GFP-LC3 were transfected with OE-MCPIP1 and with siTRAF6 or an empty vector. After 48 hours, fluorescence microscopy was used for analysis. *Scale bar*: 10 µm. Data are presented as mean ± SD; ****P < 0.0001. (**F**) The protein levels of MCPIP1, TRAF6, and LC3 in HPF cells transfected with OE-MCPIP1 alone or cotransfected with siTRAF6 were analyzed by Western blot. β-Actin was used as a control. (**G**) The plasmids encoding siTRAF6, Myc-BECN1, and Flag-PIK3C3 were individually transfected into HPF cells with either OE-Vector or OE-MCPIP1. After a 2-day incubation period, immunoprecipitation was performed on the cell lysates using an anti-Myc antibody. The resulting complexes were analyzed by immunoblotting with anti-Flag and anti-Myc antibodies. (**H**, **I**) Cell proliferation (**H**) and cell migration ability (**I**) of HPF cells transfected with OE-MCPIP1 or cotransfected with siTRAF6 were assessed. Data are presented as mean ± SD; ns, not significant; **P < 0.01, ****P < 0.0001.

TRAF6 functions as an E3 ubiquitin ligase, mediating the ubiquitination of BECN1 and thereby regulating TLR4-induced autophagy.^37^ To investigate the role of MCPIP1 in this process, pterygium fibroblasts were treated with the TLR4 agonist LPS, and the impact of MCPIP1 on the endogenous ubiquitination levels of TRAF6 and BECN1 was assessed. Our findings revealed that the MCPIP1 overexpression suppressed TRAF6 ubiquitination while enhancing BECN1 ubiquitination (Figs. 6A, 6B).

To elucidate the molecular mechanism underlying the impact of MCPIP1 on autophagy, we conducted an immunofluorescence assay in OE-MCPIP1 HPFs. The results revealed that upon exposure to autophagy agonist LPS or Poly I:C, the MCPIP1 overexpression significantly enhanced the fluorescence signal of LC3 in cells. Further, the inhibition of autophagy by 3-MA markedly attenuated the fluorescence signal of LC3 (Fig. 6C). Consistently, immunoblotting analysis showed that MCPIP1 overexpression leads to an upregulation of LC3-II expression in the cells, an effect further potentiated upon stimulation with LPS or Poly I:C (Fig. 6D).

Next, to understand whether MCPIP1 regulates autophagy via the TRAF6-BECN1 signaling pathway, we transfected HPFs stably expressing GFP-LC3 with either an MCPIP1 overexpression plasmid alone or cotransfected with siTRAF6 plasmids. Cells transfected with only OE-MCPIP1 plasmid exhibited a significant increase in the punctate aggregation of GFP-LC3. However, cotransfection with siTRAF6 plasmids markedly reduced the GFP-LC3 signal (Fig. 6E). Furthermore, compared with the MCPIP1 overexpression alone, the knockdown of TRAF6 using siRNA reduced the LC3 protein levels (Fig. 6F). As a constituent of the class III PtdIns3K complex, BECN1 typically interacts with PIK3C3 to induce autophagy.^8^ Overexpression of MCPIP1 enhanced the interaction between BECN1 and PIK3C3, while this effect was attenuated by siTRAF6 (Fig. 6G). Similar trends were observed in the assessment of cell viability and proliferation in HPFs (Figs. 6H, 6I). In summary, the results suggest that TRAF6 plays a crucial role in the MCPIP1-mediated stimulation of autophagy.

### TUT7 Regulated the Stability of MCPIP1 mRNA

**Figure 7.**
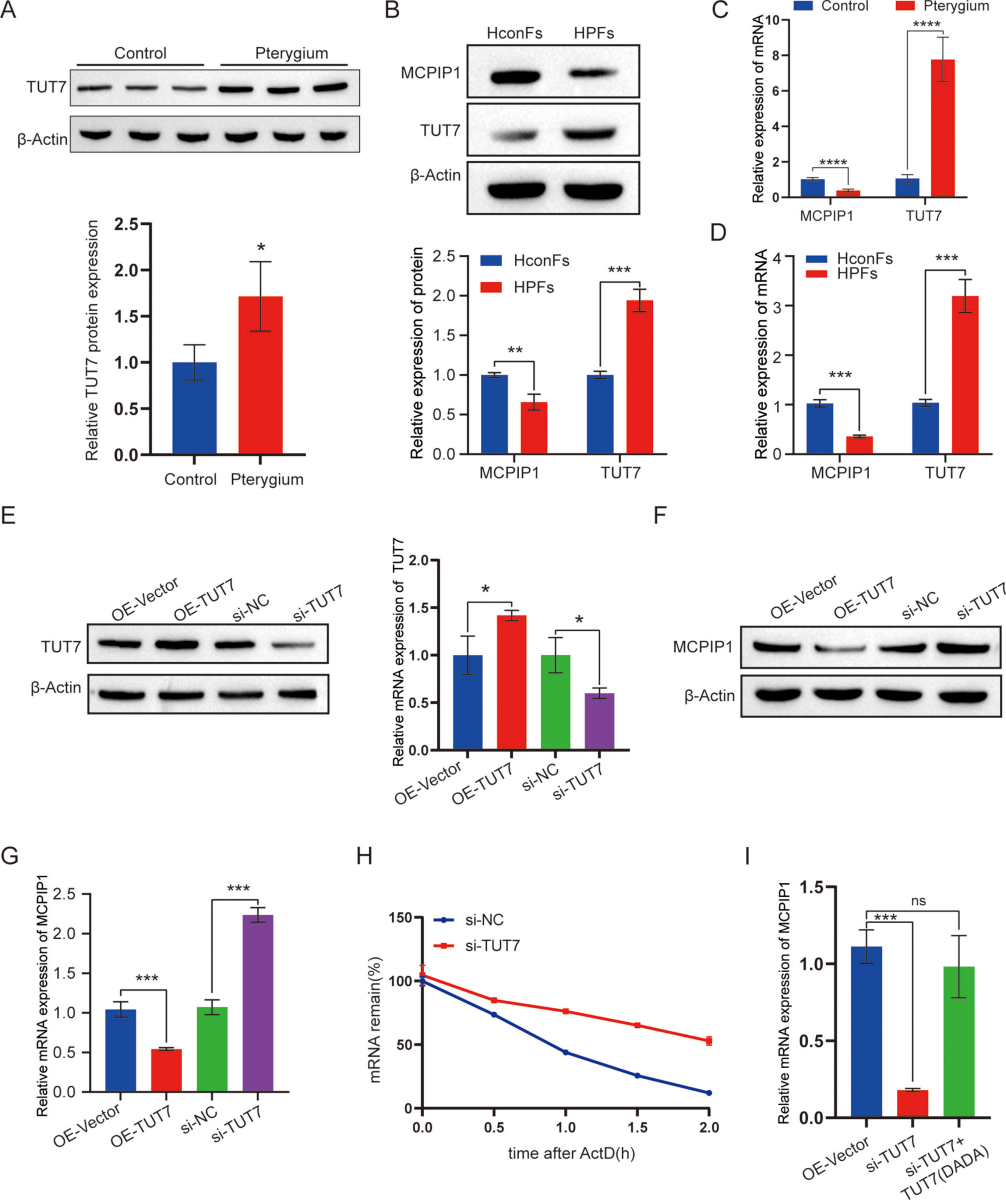
TUT7 regulates the stability of MCPIP1 mRNA. (**A**) Protein levels of MCPIP1 and TUT7 were detected using Western blot analysis in the pterygium mouse model. β-Actin was used as a loading control. Data are presented as mean ± SD; **P < 0.01. (**B**) Protein levels of MCPIP1 and TUT7 were detected using Western blot analysis in HConFs and HPFs. β-Actin was used as a loading control. Data are presented as mean ± SD; **P < 0.01, ***P < 0.001. (**C**) mRNA expression of MCPIP1 and TUT7 was detected using qRT-PCR in the pterygium mouse model. Data are presented as mean ± SD; ***P < 0.001. (**D**) The mRNA expression of MCPIP1 and TUT7 was detected using qRT-PCR in the HConFs and HPFs. Data are presented as mean ± SD; ***P < 0.001. (**E**) TUT7 protein levels were detected in the HPFs transfected with OE-TUT7 and si-TUT7 using Western blot analysis. HPFs transfected with an empty vector served as the negative control. β-Actin was used as a loading control. Data are presented as mean ± SD; ***P < 0.001, ****P < 0.0001. (**F**, **G**) The protein (**F**) and mRNA levels (**G**) of MCPIP1 were quantified in HPFs transfected with OE-TUT7 and si-TUT7. HPFs transfected with empty vectors served as negative controls. β-Actin was used as a loading control. Data are presented as mean ± SD; ***P < 0.001. (**H**) MCPIP1 mRNA levels were detected using qRT-PCR in the HPF cells transfected with si-NC, and si-TUT7 were treated with ActD (5 µg/mL). (**I**) MCPIP1 mRNA levels were detected using qRT-PCR in HPF cells coexpressing OE-TUT7 or si-TUT7 + TUT7 (DADA). Data are presented as mean ± SD; *P < 0.05, ****P < 0.0001.

Previous studies suggest that TUT7, a terminal uridyltransferase 7, facilitates the uridylylation and subsequent degradation of MCPIP1 mRNA by appending uridine at its 3′ end, thereby modulating TLR4-induced inflammation.^24^ The modification of RNA at its 3′ end through the addition of nontemplate nucleotides is an evolutionarily conserved mechanism that regulates RNA stability and fate.^38^ TUT enzymes, belonging to the noncanonical poly(A) polymerase family, play a conserved role in RNA processing from fission yeast to humans.^39^ Specifically, TUT7 (also known as ZCCHC6) is primarily responsible for cytoplasmic 3′ uridylation of various RNAs.^40^ Additionally, TUT7 triggers the oligouridylation of histone mRNA lacking a poly(A) tail at the conclusion of the S phase of the cell cycle, thereby enhancing its degradation.^24^ However, the regulatory mechanisms by which TUT7 influences MCPIP1 in pterygium remain to be elucidated. In the pterygium mouse model, TUT7 expression was found to be significantly elevated, exhibiting a negative correlation with MCPIP1 expression (Figs. 7A, 7C). Similar results were also observed in the HPF cells (Figs. 7B, 7D). Therefore, to assess the role of TUT7, we generated stable HPF cell lines with TUT7 overexpression (OE-TUT7) or downregulation (si-TUT7) (Fig. 7E). Overexpression of TUT7 suppressed MCPIP1 expression, whereas TUT7 silencing led to MCPIP1 upregulation (Figs. 7F, 7G). Moreover, in TUT7-silenced HPF cells, the MCPIP1 mRNA was prolonged (Fig. 7H), indicating that TUT7 modulates the MCPIP1 mRNA stability. The nucleotidyltransferase activity of the TUT7 critically depends on the aspartic acid residues (D) located at positions 1058 and 1060.^24^^,^^41^ To investigate the activity, a mutant variant of TUT7 with two alanine substitutions at these residues (DADA) was generated and transfected in si-TUT7 HPF cells. While TUT7 overexpression reduced the MCPIP1 mRNA level, the effect was attenuated in si-TUT7 cells expressing TUT7 (DADA) mutant (Fig. 7I). In conclusion, we proposed that the nucleotidyltransferase activity of TUT7 plays a crucial role in modulating the stability of MCPIP1 mRNA.

### TUT7 Regulated MCPIP1 mRNA Stability Via Uridylylation

**Figure 8.**
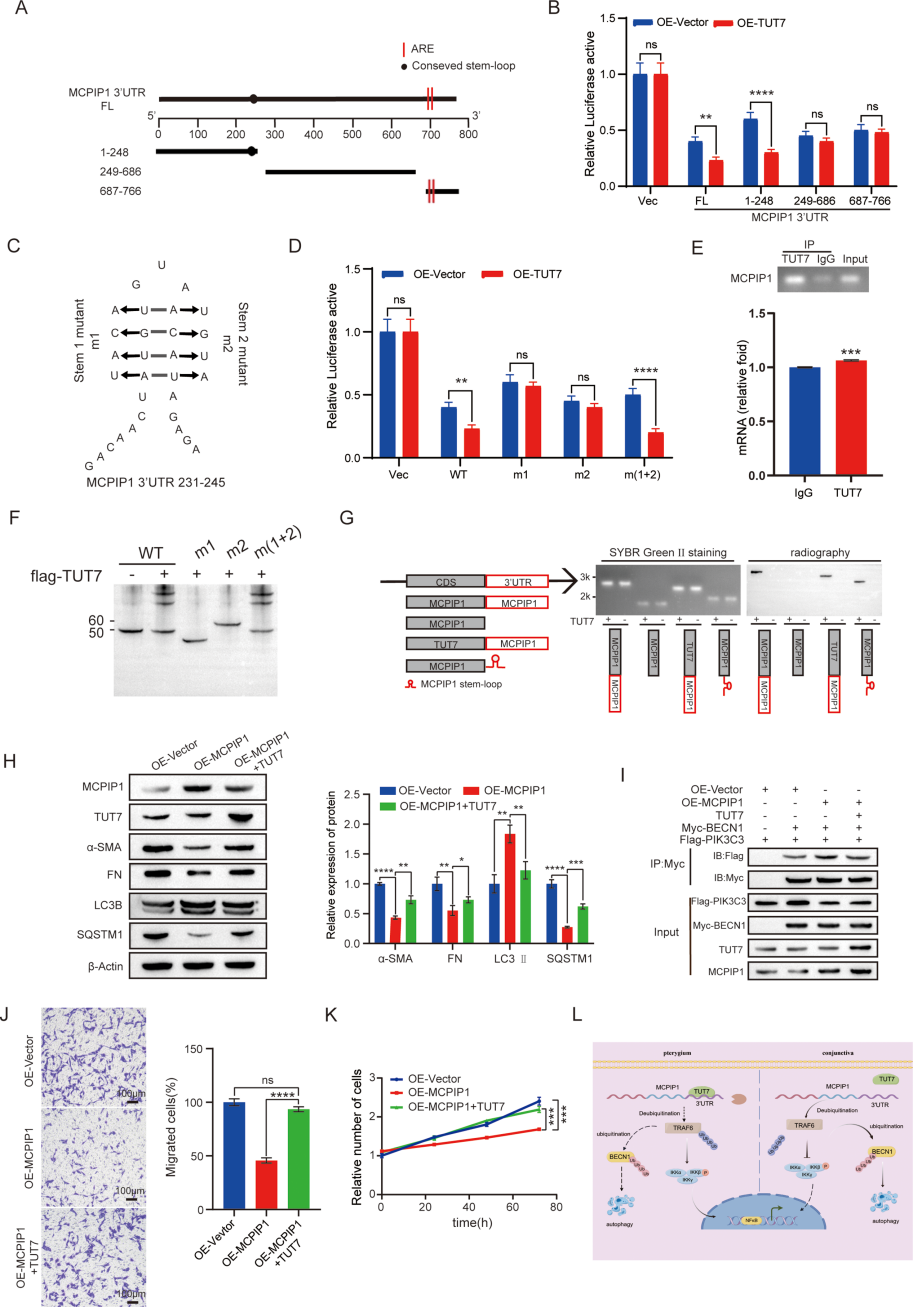
TUT7 controlled MCPIP1 mRNA stability via uridylation. (**A**) Schematic diagram of the full-length (FL) and truncated fragments of the MCPIP1 3′-UTR. (**B**) HPF cells were cotransfected with luciferase reporter plasmids containing FL or truncated fragments of the MCPIP1 3′-UTR, along with OE-Vector and OE-TUT7. Forty-eight hours after transfection, cells were collected, and luciferase activity was quantified. Data are presented as mean ± SD; ns, not significant; **P < 0.01; ****P < 0.0001. (**C**) Schematic depiction of stem-loop mutants (m1 and m2) in the 231–245 region of the MCPIP1 3′-UTR. (**D**) HPF cells were cotransfected with luciferase reporter plasmids containing MCPIP1 WT and MCPIP1 mutants with either OE-Vector or OE-TUT7. Forty-eight hours after transfection, cells were collected, and luciferase activity was quantified. Data are presented as mean ± SD; ns, not significant; **P < 0.01; ****P < 0.0001. (**E**) HPF cell lysate was incubated with anti-IgG and anti-TUT7 antibodies at 4°C for 4 hours for RNA-immunoprecipitation analysis. Data are presented as mean ± SD; ***P < 0.001. (**F**) Synthetic RNA containing WT or mutant MCPIP1 stem-loop was incubated with immunopurified Flag-TUT7 at 37°C for 30 minutes to perform the electrophoretic mobility shift assay experiment. (**G**) Schematic diagram of the different structures used for in vitro transcription (*left*). Visualization of results from in vitro uridylylation experiments using SYBR Green II staining and autoradiography (*right*). (**H**) Protein levels of α-SMAMA, FN, LC3B, and SQSTM1 in OE-MCPIP1 and OE-MCPIP1 + TUT7 HPFs cells were detected by Western blotting analysis. β-Actin served as a loading control. Data are presented as mean ± SD; *P < 0.05, **P < 0.01, ***P < 0.001, ****P < 0.0001. (**I**) The cell lysates of HPFs transfected with OE-Vector, OE-MCPIP1, TUT7, Myc-BECN1, and Flag-PIK3C3 were subjected to IP experiments using anti-Myc antibody. Immunoblot analysis was performed using anti-Flag and anti-Myc antibodies. (**J**, **K**) Cell migration (**J**) and proliferation (**K**) assays were performed on HPFs harboring OE-MCPIP1 and OE-Vector cotransfected with TUT7 HPFs. Data are presented as mean ± SD; ns, not significant; ***P < 0.001, ****P < 0.0001. *Scale bar*: 100 µm. (**L**) Model depicting the TUT7-mediated regulation of MCPIP1 and its effect on TRAF6-BECN1–induced autophagy. In the pterygium tissue, the expression of MCPIP1 induced TRAF6 deubiquitination, thereby promoting BECN1 ubiquitination and triggering autophagy to prevent pterygium development. However, overexpression of TUT7 bound to the 3′-UTR of MCPIP1 mRNA and led to its degradation, inhibiting TRAF6-BECN1–induced autophagy. Instead, it facilitated IKK complex activation and NF-κB expression, promoting pterygium progression.

To investigate the regulatory role of TUT7 in MCPIP1 mRNA via its 3′-UTR, a dual-luciferase assay was performed. We constructed a series of luciferase reporter plasmids harboring full-length or various truncated fragments of the MCPIP1 3′-UTR and quantified their luciferase activity in the TUT7-overexpressing HPF cell lines. Upon TUT7 overexpression, only cells with full-length MCPIP1 3′-UTR and MCPIP1 3′-UTR 1–248 showed a reduction in luciferase activity (Figs. 8A, 8B). Notably, the 1–248 motif of the MCPIP1 3′-UTR is predicted to form a stem-loop structure.^42^ To investigate the role of the stem-loop, mutations were introduced into the stem-loop structure, and corresponding luciferase reporter plasmids were generated (Fig. 8C). Remarkably, m (1 + 2) was generated by swapping sequences on two stems in the MCPIP1 3′-UTR, and the stem-loop mutants (m1 and m2) abolished the OE-TUT7–mediated inhibitory effect on MCPIP1 mRNA, whereas restoring the stem-loop at both positions (m1 and m2) reinstated the effect (Fig. 8D). RNA-immunoprecipitation experiments confirmed the binding of TUT7 to MCPIP1 mRNA (Fig. 8E). Furthermore, electrophoretic mobility shift assays showed that the interaction between TUT7 to MCPIP1 mRNA is dependent on the stem-loop structure (Fig. 8F). To further assess the uridylylation activity of TUT7 on MCPIP1, in vitro uridylylation experiment was performed. The results revealed that irrespective of the coding sequence (CDS) substrate being MCPIP1 or TUT7, TUT7 exclusively catalyzed uridylation on transcripts containing both MCPIP1 3′-UTR and MCPIP1 stem-loop structure (Fig. 8G). Collectively, these findings suggested that TUT7 regulates the MCPIP1 mRNA stability by uridylating the stem-loop structure within its 3′-UTR motif.

Given the high expression of TUT7 in pterygium tissue (Fig. 7), additional evidence was sought to establish its impact on pterygium progression by regulating the MCPIP1 mRNA stability. In line with this, the effects of TUT7 and MCPIP1 coexpression on fibrosis and autophagy in the pterygium were investigated. Coexpression of TUT7 and MCPIP1 led to an upregulation of α-SMAMA, FN, and SQSTM1 expression compared with the overexpression of MCPIP1 alone, while LC3-II expression was downregulated (Fig. 8H). Furthermore, TUT7 not only hindered the facilitative role of MCPIP1 in promoting the interaction between BECN1 and PIK3C3 (Fig. 8I) but also reinstated the proliferative activity of HPF cells upon the overexpression of MCPIP1 (Figs. 8J, 8K). In conclusion, the progression of pterygium is regulated by TUT7 through the uridylylation of MCPIP1 mRNA.

## Discussion

The pterygium is caused by the excessive proliferation of benign fibrovascular tissue and is characterized by cell proliferation, inflammatory response, angiogenesis, and ECM deposition.^28^^,^^43^ The etiology of pterygium is associated with multiple factors, including UV radiation, environmental influences, and oncogenic viral agents. Originating from the centripetal growth of limbal stem cells (LSCs), pterygium initiation is influenced by the UV-mediated alterations in LSCs, along with the upregulation of inflammatory cytokines, matrix metalloproteinases, and growth factors, thereby promoting inflammation, angiogenesis, and the invasion of pterygium.^44^ Additionally, human papillomavirus (HPV), a known carcinogen, has been frequently associated with pterygium. Numerous studies have identified the presence of HPV in pterygium lesions.^45^ Furthermore, a high recurrence rate of pterygium has been observed following HPV infection.^46^

The primary challenge in pterygium treatment is the fibrosis resulting from excessive wound healing after excision surgery. Therefore, fibrosis-related markers are commonly employed to characterize the progression of pterygium.^47^^,^^48^ In this study, α-SMA and FN expression was significantly upregulated in the MAHPF cells isolated from human pterygium tissue, exhibiting the typical fibrotic morphology. These results are consistent with the aforementioned argument. Notably, MCPIP1 (also known as ZC3H12A), characterized by the presence of CCCH Zn finger domains, is a known regulator of fibrosis in different tissues. MCPIP1 modulates the migration of fibroblasts within a three-dimensional collagen matrix.^49^ It also facilitates the SiO_2_-induced enhancement of migration in lung fibroblasts during silicosis pathogenesis.^50^ Our research findings suggested that MCPIP1 was downregulated in the pterygium mouse model, suggesting its negative regulatory role in pterygium fibrosis. As shown in Fig. 8L, MCPIP1 inhibited fibrosis progression by enhancing the interaction between TRAF6 and BECN1 and promoting BECN1 ubiquitination. The ubiquitinated BECN1 further triggered cellular autophagy, thereby modulating cell migration and proliferation. Furthermore, TUT7, by binding to the 3′-UTR region of MCPIP1 mRNA, negatively regulated MCPIP1 expression through uridylylation, influencing downstream effects.

MCPIP1, previously identified as a deubiquitinase, targets the TRAF family, thereby regulating the JNK and NF-κB signaling pathways.^31^ The activation of the NF-κB signaling pathway was also observed in pterygium.^51^ TRAF6, the E3 ubiquitin ligase, undergoes K48-linked self-ubiquitination, thus facilitating the activation of the NF-κB signaling pathway.^21^^,^^36^ TRAF6 promoted the K63-linked ubiquitination of BECN1, thereby modulating the assembly of the BECN1-PIK3C3-PIK3R4 complex, which was crucial for autophagy initiation.^21^^,^^35^^,^^52^^–^^55^ Autophagy receptor proteins, such as SQSTM1/p62, possess the ability to recognize degradation signals on autophagic substrates and facilitated the localization of LC3 on expanding autophagosome membranes.^4^^,^^56^ The assessment of the autophagy level commonly relies on the utilization of SQSTM1 and LC3. In this study, MCPIP1 overexpression induced the upregulation of LC3 protein expression and concomitantly downregulated SQSTM1 receptor protein levels, indicating robust autophagic activity. We observed a direct interaction between MCPIP1 and TRAF6, which induced the deubiquitination of TRAF6. Notably, the NCR and ZN domains of MCPIP1, along with the TRAF-C domain of TRAF6, were found to be crucial for mediating this interaction. The NCR and ZN domains of MCPIP1, along with the TRAF-C domain of TRAF6, played pivotal roles. Also, the existence of the TRAF6-BECN1 signaling cascade was confirmed. Based on this, we observed an interaction between the Zn domain of MCPIP1 and the coiled helical structure of BECN1. However, the ubiquitination level of BECN1 remained unchanged even with increasing doses of MCPIP1. The MCPIP1 protein facilitated the interaction between TRAF6 and BECN1, thereby augmenting the ubiquitination modification of BECN1 by TRAF6. This process further stimulated the assembly of the BECN1-PIK3C3 complex, inhibiting migration and proliferation in HPF cells. The regulation of autophagy by MCPIP1 was impeded by siTRAF6, suggesting that the activation of autophagy by MCPIP1 relies on TRAF6. The dynamic regulation of autophagy levels plays a crucial role in the fibrotic process across various organs. Sustained elevation of autophagy in renal tubular cells triggered the release of profibrotic factors, thereby promoting kidney fibrosis.^57^ The decrease in autophagy induced by TLR4 was accompanied by a reduction in the degree of pulmonary fibrosis.^16^ In the context of pterygium, autophagy exerted inhibitory effects on the SQSTM1–NF-κB signaling pathway through the degradation of the SQSTM1 protein, thereby exerting negative regulation on fibroblast fibrosis and subsequently impacting the progression of pterygium.^4^ However, further investigation is required to elucidate the precise mechanism through which autophagy modulates the progression of pterygium.

Polyadenylation and uridylylation at the 3′ terminus of RNA represent the pivotal mechanisms governing RNA stability. The former serves to augment transcript stability, while uridylylation functions as a degradation marker for RNA.^38^ TUT4 and TUT7, members of the TUT family, are terminal nucleotidyltransferases classified as atypical poly(A) polymerases. They play a crucial role in uridylylation modification at the 3′ end of diverse RNA molecules, including precursor microRNAs, histone mRNAs, and noncoding RNAs, thereby exerting control over RNA stability.^58^^–^^60^ TUT4/TUT7 can catalyze the uridylylation of miRNA precursors, resulting in either the biogenesis or degradation of miRNA.^58^ According to reports, TUT7 plays a role in stabilizing IL-6 expression in chondrocytes by uridylylation and stabilization of miR-26b.^61^ TUT7 exerted regulatory control over IL-6 expression by modulating the stability of MCPIP1 mRNA.^24^ These studies provided evidence for the involvement of TUT7 in RNA degradation via uridylylation. Our research revealed a significant upregulation of TUT7 in both the pterygium mouse model and HPF cells, exhibiting a strong negative correlation with MCPIP1 expression. Furthermore, the overexpression of TUT7 resulted in near-complete suppression of MCPIP1 expression. However, after TUT7 knockdown, the half-life of MCPIP1 mRNA was prolonged. The nucleotidyltransferase activity of TUT7 was crucial for its uridylylation modification.^41^^,^^59^ Similarly, we observed that the abrogation of nucleotidyltransferase activity in TUT7 led to the loss of its regulatory function on MCPIP1 mRNA. Recent studies have demonstrated that TUT7 specifically interacts with the stem-loop structure located in the 3′-UTR of MCPIP1, thereby facilitating uridylylation and subsequent degradation of ZC3H12A.^24^^,^^42^ Consistent with these findings, we also observed that TUT7 specifically targeted the stem-loop structure of MCPIP1 and exerted inhibitory effects on its 3′-UTR activity. Ultimately, this repression hampered autophagy while promoting cell migration and proliferation, thereby contributing to fibrosis in the pterygium.

In summary, this study elucidates the potential molecular mechanism by which MCPIP1 regulates the TRAF6-BECN1 signaling pathway and influences autophagy activation and pterygium progression.. Furthermore, our research offers a novel direction for investigating the role of uridylylation modification in disease progression. While the study provides valuable insights into the molecular mechanisms of pterygium progression and potential therapeutic strategies, the precise mechanism by which autophagy influences pterygium fibrosis has yet to be elucidated. Furthermore, the findings of this study are currently unsupported by data from clinical trials. Overall, the results of our research provide valuable insights into the pathological mechanisms underlying pterygium and offer innovative strategies for preventing its recurrence in treatment plans.

## Supplementary Material

Supplement 1

Supplement 2

Supplement 3
