# Supplementary material for: TUT7-Mediated Uridine Degradation of MCPIP1 in the Pterygium to Regulate TRAF6-Mediated Autophagy
Source: Invest Ophthalmol Vis Sci. 2025 Apr 16;66(4):41. doi: 10.1167/iovs.66.4.41 (PMC12011128; doi:10.1167/iovs.66.4.41)
Supplement: Supplement 1 [file iovs-66-4-41_s001.pdf]

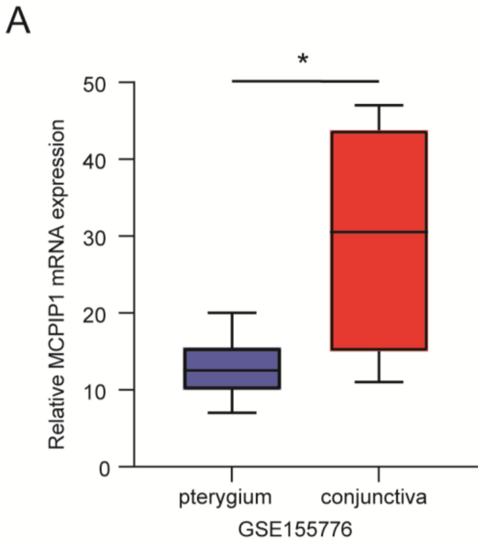

Figure S1. MCP1 is expressed at a low level in pterygium

(A) Boxplot of the MCPIP1 expression analysis in the GSE155776 dataset. *P < 0.05.
