# Supplementary material for: TUT7-Mediated Uridine Degradation of MCPIP1 in the Pterygium to Regulate TRAF6-Mediated Autophagy
Source: Invest Ophthalmol Vis Sci. 2025 Apr 16;66(4):41. doi: 10.1167/iovs.66.4.41 (PMC12011128; doi:10.1167/iovs.66.4.41)
Supplement: Supplement 2 [file iovs-66-4-41_s002.pdf]

Supplementary Table 1 Characteristics of the patients and donors

Number	Gender	Age	Specimen	Surgery Date	Operation start time	Operation end time
P1	Female	36	Pterygium of the left eye	2024.5.16	11:20	12:00
P2	Male	55	Pterygium of the left eye	2024.6.12	14:45	15:30
P3	Male	62	Pterygium of the right eye	2024.6.18	12:25	13:00
P4	Female	55	Pterygium of the left eye	2024.6.24	13:50	14:30
P5	Female	54	Pterygium of the right eye	2024.7.17	13:30	14:00
P6	Female	58	Pterygium of the left eye	2024.7.25	11:20	11:45
P7	Male	42	Pterygium of the right eye	2024.7.26	11:15	11:40
P8	Female	43	Pterygium of the right eye	2024.7.30	13:45	14:20
P9	Male	57	Pterygium of the left eye	2024.8.7	11:15	11:50
P10	Female	65	Pterygium of the right eye	2024.8.8	18:15	19:00
P11	Male	51	Right eye superior bulbar conjunctiva	2024.3.28	15:10	16:15
P12	Male	40	Right eye superior bulbar conjunctiva	2024.4.7	13:00	14:20
P13	Female	69	Right eye inferior bulbar conjunctiva	2024.4.9	16:30	18:00
P14	Female	50	Right eye superior bulbar conjunctiva	2024.4.26	13:00	14:05
P15	Male	46	Right eye superior bulbar conjunctiva	2024.5.10	12:50	14:10

P16	Female	66	Right eye inferior bulbar conjunctiva	2024.6.19	13:40	15:05
P17	Female	46	Left eye superior bulbar conjunctiva	2024.6.26	12:20	14:00
P18	Female	61	Right eye superior bulbar conjunctiva	2024.6.27	9:15	10:30
P19	Male	32	Left eye superior bulbar conjunctiva	2024.7.10	11:15	12:50
P20	Male	35	Right eye superior bulbar conjunctiva	2024.7.25	13:20	15:05
