# Supplementary material for: TUT7-Mediated Uridine Degradation of MCPIP1 in the Pterygium to Regulate TRAF6-Mediated Autophagy
Source: Invest Ophthalmol Vis Sci. 2025 Apr 16;66(4):41. doi: 10.1167/iovs.66.4.41 (PMC12011128; doi:10.1167/iovs.66.4.41)
Supplement: Supplement 3 [file iovs-66-4-41_s003.pdf]

Supplementary Table 2 The primer sequences of qRT-PCR

	Forward (5’-3’)	Reverse (5’-3’)
MCPIP1-Mouse	CTGGAGAGCCAGATGTCAGAATTA	GTACTCTCTGGATGGGTAGGTGG
MCPIP1-Human	CTGGAGAAGAAGAAGATCCTGG	TGACGAAGGAGTACATGAGCAG
TUT7-Human	GAAGAGAAGGGCACATTAAAAAGG	GGAAAGGCTACCTGAAGAGC
TUT7-Mouse	ACCTGTGTTTCAGACCCTTAC	CAGCTGCATTTCCCAGTTTATC
GAPDH-Human	GATTCCACCCATGGCAAATTC	CTGGAAGATGGTGATGGGATT
GAPDH-Mouse	CATCACTGCCACCCAGAAGACTG	ATGCCAGTGAGCTTCCCGTTCAG
